# Gender, Race, and Immigrant Status: Intersecting Implications for Health in Middle and Later Life

**DOI:** 10.1093/geroni/igaa057.1094

**Published:** 2020-12-16

**Authors:** Margaret Penning, Neena Chappell, Sean Browning, Helena Kadlec

**Affiliations:** University of Victoria, Victoria, British Columbia, Canada

## Abstract

Although the negative implications of gender, race and immigrant inequalities for health and well-being in the middle and later years of life are well-documented, there is a lack of research addressing their combined implications as well as the mechanisms linking them to various health-related outcomes. Yet, as intersectionality theory reminds us, the consequences of gender, race, immigrant and other inequalities for physical and mental health outcomes must be understood in terms of these overlapping social identities. Moreover, linking intersectionality to stress process theory provides us with an explanation of the mechanisms potentially linking intersecting structural inequalities to health outcomes. This paper draws on data from the Canadian Longitudinal Study on Aging (CLSA - N=51,338) to assess the additive and interactive implications of gender, race and immigrant status for physical and mental health outcomes, together with the mediating effects of primary and secondary stressors on these outcomes. The results of a series of weighted least squares regression analyses suggest that immigrant status interacts with race and/or gender to influence health outcomes. Socioeconomic and other stressors also play a role in linking these intersecting structural inequalities to health outcomes. Overall, our findings provide initial support for the value of linking intersectionality and stress process frameworks for an understanding of the health implications of structural inequalities in middle and later life.

